# Understanding the Narratives of Child Sexual Abuse

**DOI:** 10.1177/10497323231218828

**Published:** 2024-01-16

**Authors:** Mari Dalen Herland

**Affiliations:** 1 Social work, 87368 VID Specialized University, Oslo, Norway

**Keywords:** child sexual abuse, qualitative interviews, narrative analysis

## Abstract

This qualitative study consisted of in-depth, retrospective interviews with participants over the age of 18 who experienced child sexual abuse. Through narrative analysis, study findings shed light on three overall findings: the perception of memories, the perception of grooming, and the perception of the lived lives of child sexual abuse survivors. Findings suggest that the narratives elicited from the participants included current views but also past experiences and anticipation about the future, including individual and societal levels of meaning. These narratives are furthermore entangled and inexorably linked – temporally, culturally, generationally, materially, and emotionally – and the results are thus presented from a holistic perspective. Study findings help explain the complex dimensions concerning the lived experiences of child sexual abuse. As such, this research speaks to the field of social and health care practitioners working with children and families facing the complex phenomenon of child sexual abuse.

## Introduction

Child sexual abuse is viewed as a physical assault upon the body and as a complex traumatic experience affecting both body and mind (Daphna-Tekoah, 2019; Felitti, 1991, 2019; Van der Kolk, 1994). Studies acknowledge the long-term psychological and physiological effects of adverse childhood experiences, such as child sexual abuse (Felitti, 1991, 2019). However, child sexual abuse is a complex phenomenon, compounded by many individual and sociocultural factors (Alaggia, 2004, 2005). Hence, the aim of this article is to explore the dimensions the participants emphasized in their narratives, using retrospective, qualitative, in-depth interviews. This research contributes to the field of social and health care practice, especially services that support children and families facing the phenomenon of child sexual abuse.

Further knowledge is needed on the more complex, problematic elements of child sexual abuse, with a holistic view. The article explores the personal and social aspects of narratives from individuals who have experienced child sexual abuse; it sheds light on the challenges they face and the realities they encounter, both within public discourse and through their own ability to share and articulate their experiences (Cooper, 2009; Etherington, 2004).

### A Public Health Problem

According to the Convention on the Rights of the Child, state parties are obliged to undertake actions to protect children from all forms of sexual exploitation and abuse. The World Health Organization (1999) defines child sexual abuse as the involvement of a child in sexual activity that they do not fully comprehend, are unable to give informed consent to, or are not developmentally prepared for, or that violates social norms. Research indicates that about one in eight people will experience child sexual abuse globally; it represents a significant public health problem, with up to 17% of boys and 31% of girls experiencing child sexual abuse worldwide (Lange et al., 2020). Child sexual abuse is thus an extensive public and social health concern (Collin-Vézina et al., 2021).

Research has further shown that children often do not disclose abuse until they are adults (Priebe & Svedin, 2008). Reasons for not disclosing include lack of awareness or understanding, fear of being disbelieved and blamed, perceived responsibility, a close and multifaceted relationship with the perpetrator, and fear of negative consequences for themselves or other family members (Alaggia, 2004; Collin-Vézina et al., 2021; Winters et al., 2020). The immediate and long-term negative effects of child sexual abuse have been well documented and include emotional, cognitive, behavioural, interpersonal, and physical health consequences (Berliner & Elliott, 2002). Research has also revealed the need to improve professionals’ capacity to help children who have experienced child sexual abuse (Albaek et al., 2020). This points to the complexities entailed by this phenomenon, and therefore, more knowledge is greatly needed. Drawing on participants’ own reflections on how they perceive their lived experiences, this article provides valuable knowledge for practitioners to inform their public health practice as will be discussed further.

## Objectives

The main objective is to enhance understanding around the experiences of individuals who have faced child sexual abuse. This includes exploring how they demonstrate agency, as well as their ability to resist and transform their lives in light of these experiences. The project’s aim is thus to generate knowledge that can be utilized by health and welfare institutions to provide better assistance and care for those affected.

## Theoretical Orientation: Relational Ontology and a Holistic Point of View

Recently, there has been increasing discussion across the social sciences about an ‘ontological turn’ (Spyrou, 2019). In terms of thinking relationally about children’s experiences (Spyrou et al., 2018; Spyrou, 2019), such as child sexual abuse, relational ontology invites a relational stance not only towards bodies and persons but also objects, technologies, systems, epistemes, historical eras, and temporal dimensions (Spyrou et al., 2018; Varpanen, 2019). This ontological turn can be understood as a reaction to the linguistic/cultural turn of the ‘90s. While the latter turned attention to issues of language, discourse, and representation, the former seeks a return to the ‘real’ and the materiality of life. As such, an ‘ontological turn’ promises a shift from issues of epistemology to issues of ontology, where relationality and materiality become central features in our ongoing attempts to understand the world and our place in it. What the ontological turn offers is the theoretical possibility of recognizing the materiality of life while understanding the discourse with which it is entangled (Spyrou et al., 2018; Spyrou, 2019; Varpanen, 2019).

In this article, by ontology, I mean understanding the participants’ narratives as connected to various aspects of their lives, such as time, relationships, context/culture, and material circumstances. This includes considering their experiences within a specific historical era or generation, as well as the places and spaces they navigate. Thus, the units of analysis in this article are based on an understanding of the participants as ontological becomings: What matters is not what they are or were, but how they affect and are affected by the assemblages in which they found/find themselves, and the resources available (Spyrou et al., 2018; Spyrou, 2019). In other words, to be located within the framework of a broader relational turn in the social sciences (Dépelteau, 2013) means embracing a posthumanism orientation that shifts the focus of analysis to the larger networks of ‘forces’ – material, discursive (Braidotti, 2017), and emotional – as part of a holistic departure.

## Materials and Methods

This study employed a qualitative design, consisting of in-depth, retrospective interviews and a second interview 6 months later, with 14 Norwegian participants over the age of 18, some of whom could be classed as part of a minority group based on their ethnicity. The sample consisted of 2 who identified as men and 12 who identified as women. All participants lived with sexual abuse for multiple years as they were growing up, ranging from the age of 2–15. The perpetrator was their father, uncle, brother, grandfather, or a person hired by child protective services. A second interview was conducted to follow up with certain themes that needed elaboration. To recruit participants, flyers with information about the project were posted online via social media and distributed to different support centres for survivors of sexual abuse. Two pilot interviews were conducted to evaluate the interview guide and the terms used in the interviews: for example, ‘victims’ versus ‘survivors’ was queried, as was how to initiate the interview in a sensitive way. Adjustments were then made to the interview guide (Herland, 2022a, 2022b, 2023).

In the interviews, participants were asked questions about their abusive experience, their relationships, their thoughts, and their feelings, following a semi-structured interview guide that allowed them to speak freely. Each interview lasted approximately 2 hours. Once each interview was complete, they were transcribed verbatim by the author and a qualified professional hired by the project. Identifying information was removed from the transcript and participants were given pseudonyms. The transcripts were later translated into English. For ethical reasons and privacy concerns, the data set cannot be shared.

All participants joined voluntarily. They reached out to the author after reading about the project in support centres or online. Moreover, all the participants seemed motivated to contribute, sharing rich descriptions of their experiences during the interviews. All participants commented that their participation generated positive feelings about being able to spread awareness of child sexual abuse, possibly helping others (Herland, 2022a, 2022b, 2023).

### Narrative Analysis and Reflexive Inquiry

‘Narrative’ refers to an array of approaches that reflect different theoretical and methodological orientations (Riessman, 2008). In general, the approaches attend to how people make meaning of their past and present lives, often presented within a temporal ordering of events and actions (Riessman, 2008). During the analytical step of reading through the material, attention was directed early on towards how the participants appeared to make meaning of their lived lives of child sexual abuse disclosed in a storied form, which seemed to be framed within larger stories (Gergen, 2001; Riessman, 2008). The analytical questions regarding their experiences raised questions about their spatial, temporal, and relational boundaries. This narrative analytical approach offered a frame to capture the participants’ complex lived experiences (Missel et al., 2021). A narrative approach incorporates not only the psychological work subjectively but also the social and cultural aspects of meaning-making. As a theoretical and analytical term, ‘narrative’ concerns how a story is told in a particular way, for whom it is constructed, and for what purpose: attending to what cultural resources the narrator draws upon and takes for granted, and what the story accomplishes (Riessman & Quinney, 2005).

‘Lived experiences’ refers to the phenomenological tradition concerning experiences of the everyday lifeworld. Such experiences are pre-reflective and therefore less available to one’s awareness (Galagher & Zahavi, 2012). The data were initially analyzed by the author and then presented to other researchers in research groups at the Faculty of Social Work. The analysis was developed further, based on several rounds of analysis and discussion around the narratives that surfaced. Temporal, relational, and contextual aspects stood out in the analysis. In ‘storying’ their lives, the participants appeared to attribute meaning to these life stories, especially through three narratives regarding the phenomenon of child sexual abuse. The analysis brought attention to both individual and social collective levels of meaning. I presented the analysis to one participant, who provided valuable feedback. Following Mattingly (1998), Bartoszko (2021), the participants appeared to locate themselves in unfolding grand stories of what was possible, but also anticipating how things could unfold.

In analyzing the narratives, I was attuned to their content, but also the contexts in which the events happened: contextual, relational, temporal, societal, and cultural. I also tried to identify my own feelings and in what way these affected me as a researcher and in turn the analysis. As the participants’ stories were re-told by me and thus inherently co-constructed, this necessitated that I pay specific attention to my own subjectivity in the re-telling, to examine the ways in which ‘the author’s meanings’ may be at work in the new narrative (Herland, 2022a, 2022b, 2023). Sharing my reflections with two members of a reference group had a valuable impact.

Through the literature review and initial phase of the project, I became aware of the importance of addressing issues of retraumatizing factors, primarily in relation to the participants but also to myself as a researcher. I therefore examined my position reflexively, looking into my own emotional responses. I reflected on how the interviews affected the participants but also me. I asked myself why I was affected, and again questioned how this impacted the interviews, the participants, and the analytical process – and further, the research outcomes. I wrote down my thoughts, feelings, and embodied sensations. I took notes immediately after the interviews, as I found that I felt strongly empathetic towards all the participants during the interviews and that, afterwards, I would dream about their stories. I could feel in my body an anxiousness, as if I could relate bodily to their difficult experiences, although I had not experienced child sexual abuse. This was peculiar and surprising (Herland, 2022a, 2022b, 2023). These concepts and experiences fostered a heightened acknowledgement of the narrator–audience interplay, as well as the interconnection between the individual possessing knowledge and the information being acquired. Every participant in the research relationship thus brought their unique perspectives and experiences, contributing to the creation of meaning and comprehension in the analysis (Herland, 2022a, 2022b, 2023).

### Ethical Considerations

From an ethical standpoint, maintaining a focus on the participants’ emotional well-being and treating the participants and the material with respect were critical. All the participants were in therapy or had ended therapy at the time of the interview. Throughout the study, the participants were followed up with on several occasions, given information regarding where to seek professional assistance if needed, and offered help seeking such support. The participants all shared positive feelings about contributing with their experiences. However, their well-being was a serious ethical concern throughout the research project and was discussed within both the project team and the research group at the Faculty of Social Work. I also followed up with the participants after a period of time, again advising them where to seek help if needed. The participants reported that contributing to the research project felt meaningful and that they hoped the results would promote societal awareness regarding child sexual abuse.

The study was approved by the Norwegian Agency for Shared Services in Education and Research (SIKT) before the interviews were conducted. The participants also gave informed consent before both interviews (Herland, 2022a, 2022b, 2023). Throughout the research process, other ethical dilemmas were continually discussed with colleagues: for example, how to ask openly but appropriately about participants’ experiences, discussing the possible consequences of sharing past experiences, how to follow up with participants afterwards, and how to present the data with integrity and respect. The interview questions were carefully developed, and as noted earlier, participants were asked about their preferred terms (e.g. ‘survivor’ vs. ‘victim’). The analysis was also discussed with participants to validate the findings.

## Findings

When storying their life experiences, as people get woven into reflections on what stories to tell and how to tell them, the stories told should be understood as transformed, negotiated, and meaning-making experiences (Riessman, 2008). The theoretical and analytical focus in this article explores the lived experiences of individuals who grew up with child sexual abuse, presented through three overall narratives: (a) the perception of memories, which speaks to the participants’ temporality connected to the memories and emotions within their narrative; (b) the perception of grooming, which speaks to relational and material aspects; and (c) the perception of the lived lives of child sexual abuse survivors, which speaks to their insights and the contextual elements that appear to inform their notions concerning the child sexual abuse experiences. Together, these three storylines, as part of participants’ narratives of abuse, appear to be vital in their process of taking ownership of their experiences and as an act of agency in the present, where they are more in control – in contrast to what they experienced growing up.

## The Perception of Memories

In their narratives, several of the participants emphasized their memories, in order to make meaning of their experiences. These memories are intricately linked to the temporal aspect and constitute an extensive thread in this narrative. Lise explained her memories in a temporal framework in this way:

In other words, I have somehow found a way to keep it at a distance, and then live with it in a way. And then I had a real, real flashback when my first child arrived. And it was a girl. And it was at least 8–10 months before I felt it was okay to wash her underneath and stuff like that. He [the father] noticed that there was something, but I made up excuses. Because he knew nothing then.

Like several others within the group, Lise had consciously endeavoured to repress her memories, confining them to a safe distance within the recesses of her consciousness. Manifestly, the temporal aspect assumes considerable importance within this particular context. The memories were perhaps too painful to deal with, and she had found a way to handle them. However, the recollections were intrusive, especially when she had her first child; she spoke of having vivid images of the birth of her first child, followed by the abusive memories that were evoked. The temporal aspect is a significant part of her narrative: Her need to keep the memories at a distance appears to have been vital to her, and this constitutes valuable knowledge for professionals working with those exposed to abuse. The need to handle one’s memories in one’s own time frame appears to be central, indicating that professionals need to be sensitive to the temporal aspects concerning memories and the lived experiences of abuse.

Marie presented a slightly different experience, but with some similarities to Lise, as she discussed her unexpected reactions. This shows how the temporal nature of these experiences poses challenges and calls for sensitivity, in various aspects of the lives of individuals having experienced child sexual abuse:

So, I have no memories before that. But I’m a bit surprised at the reaction I had … But I have no memories that indicate that anything has happened before. But of course, one wonders. But it is the first incident that I remember very well and have clear memories of. And it is a question of how much detail I should go into.

Marie recalled experiencing vivid images, which allowed her to connect memories to a certain time – memories that were previously suppressed or unavailable to her. She reflected back on the passage of time, and for her this was a crucial factor. Regardless of whether her memories were accessible or not, the crucial point was that she had control over the temporal aspect, and it seemed that this was vital to her. In the above quote, she wonders whether she had not yet been ready to face the hidden stories her body was carrying and whether her mind helped her by keeping them locked away for a time. Marie’s reflections provide insights into how this temporal process unfolds and how important it might be for practitioners to help those who have experienced child sexual abuse maintain ownership – as to when they decide to share and when to seek professional help. Moreover, although they lacked agency in their childhood, being coerced by the adults around them, their memories are their own, and this should therefore be recognized by practitioners.

Karl explained his first memories of abuse and how he made meaning of the abuse, reflecting retrospectively. In his narrative, Karl recollects a specific moment when he comprehended what was happening to him. He also recalls that his younger sister and brother were too young to understand. Furthermore, he vividly remembers being given candy, and the materiality and temporal aspect connected to these memories stood out:

I remember getting a lot of candy. I got more than my siblings, especially my sister then. She is only one year younger than me. I have a brother who is five years younger, so he was too small then.

Recalling his memories in adulthood, Karl reflected around the candy being given and the point of time in which he was being abused. Similar to Lise, Karl had made a deliberate effort to suppress his memories, keeping them securely tucked away in the depths of his mind. Evidently, the element of time holds great significance in this specific situation. He was making meaning around the experiences and how they started, and furthermore talked about his siblings and that he felt responsible for protecting them, as he was the oldest:

So, I was very concerned about my sister. Because it was probably the first time that we … I was very concerned that my sister should not be exposed. So, I had to really look after her.

Karl’s memories of age, the candy, and his siblings are all related to the context in which the abuse happened and stand out as being of key significance, connected to material and temporal aspects. The temporal component appears to be inexorably linked to his memory, feelings, and reflections around the time of abuse.

## The Perception of Grooming

Another storyline and grand narrative that was brought forwards in the interviews was the participants’ reflections on their personal experience with grooming. They defined it as feeling trapped – primarily relationally – but also in terms of places and spaces. They all appeared to find themselves in an unsafe place, with no viable means of escaping the abuse. Their perceptions of the grooming process appear to be something they had in common and now wanted to share. Camilla explained her understanding of her lived experience of being groomed in this way:

He was very kind and straightforward at first. He would always look out for you and was very like, ‘I will always be here for you’. Gave you a lot of peace of mind. And then I trusted him even more. And then it gradually started with something like that. He said ‘Yes, when you become a big girl, you will get a boyfriend’. And then he actually started talking about it sexually. And then gradually then … Then the first episode was when he took me alone on a boat trip. Because we had a cabin on the same island. And then he drove the boat far out. We were going fishing, was what he said. But it wasn’t fishing. It was me alone on a boat with him out at sea. No one else around. And he started touching me in places he wasn’t supposed to. He actually served me alcohol.

Camilla brought forwards reflections of being subjected to a grooming relationship. This was a shared narrative that appeared to contribute to her meaning-making process, which was a commonality among most of the participants. In material terms, she realized – as part of her narrative – that she was brought onto a boat and then trapped. She was caught both materially and relationally: the place she was in, the spaces around her, and her boundedness to the perpetrator. Initially, she explained that she had begun to trust the perpetrator, who had made her believe that he was a trustworthy adult, and then this changed. In their narratives of the grooming processes that stood out in the material, participants regained agency by recognizing that they were bounded to their perpetrator, tied to the place in which they grew up, and entangled in the relationships, boats or households, and time of year of which they were a part – as an ontological becoming.

Tina explained her understanding of the grooming process and how time, place, and space were crucial, and that the relationship in which she was entangled stood out to her and appeared to be intertwined:

He never said that when he talked about sex. He told me very early on how it worked. I didn’t understand much, but I understood that it was something that people my age shouldn’t know then. Instinctively, I knew I must not tell. Things like that you mustn’t say to anyone. But otherwise, he never said not to tell or made threats or anything like that. He never did.

In Tina’s narrative about the perpetrator, similar to those of other participants, she emphasized that during that period she had a limited understanding of what was happening; however, in the present, she was able to make sense of it. Apparently, also here the element of time holds great significance. In addition, Tina recognized her instinctual awareness of the emotions, actions, and relationship dynamics. The interconnectedness was out of her control at that time. Tina’s retrospective reflections depict the perpetrator’s actions as he groomed her, thus providing a vivid rendering of her lived experience of abuse. Tina and the other participants were taken back to their earlier experiences, emotionally and embodied: Making meaning in the present, in the interview, may have helped them regain their agency.

Camilla, too, said that she had a limited understanding of what was happening back then, on the boat. She viewed these experiences from her past, in the present:

And I was five or six years old. Then I remember afterwards, when he had finished what he had done. After that he drove the boat back to the island. He followed me back to the cabin. And it was just as if nothing had happened. And I was so young that I didn’t understand what had happened. I realized it wasn’t right.

Camilla described her confusion and the entangled aspect of the grooming process, being only 5 or 6 years old and thus entirely dependent upon the adults around her, and this is how she remembered it in the present time. However, she was also aware of the materiality of being trapped on the boat and then taken back to the cabin, and how she did not comprehend what had happened. As a child, she said she knew that it was wrong but lacked the language to articulate what had happened; instead, it was captured within her body and mind as something wordless, until later, when she gradually began to understand – and this process is what she could take ownership of. She explained:

Yes, it is. There was something in me that realized it wasn’t right. He didn’t say it literally, but I understood it from the way he spoke to me. That this should be our little secret. And he didn’t actually say it verbatim. Because he had built up a lot of trust. What he did to me, it had to be right. Because I trusted him. But I also felt that it was wrong. So, it turned out that he didn’t have to say that I wasn’t allowed to say it. He didn’t need to be threatening because he had won my trust.

Camilla reflected around the concept of trust and depicted a range of emotions and thoughts regarding the grooming process. She described feelings of being bound to the perpetrator. In a material sense, she was trapped in a dangerous environment, with no apparent means of escape.

The participants’ understandings of grooming were shaped by their upbringing in a specific era, entangled in various environments. As young children, as relational becomings, they lacked the cognitive capacity to fully comprehend the relational dynamics at play and only realized later the true nature of what was happening to them. However, in their present narratives, the aspect of grooming was emphasized, captured within their minds and bodies – often with the retrospective sense that it was something confusing, wrong, or bad at the time it happened.

## The Perception of the Lived Lives of Child Sexual Abuse

The final narrative explains the participants’ notions of child sexual abuse. These perceptions of abuse are contextual and cultural, manifested in diverse ways within the data. The participants had both different and similar ways of making meaning around these experiences: some looked back at their upbringing, relating the abuse to societal views, including taboos and stigma, while others made meaning around their experiences through their therapeutic processes and a therapeutic language. Their notions of child sexual abuse, however, all reflected a certain cultural, contextual frame with which they negotiated. In accordance with aspects such as the perpetrators’ generation – the norms ‘back then’ – they also drew on contemporary views of child sexual abuse. Moreover, these narratives stood out as complex illustrations of the lived experiences of abuse. Laura, for example, talked about her experiences in this way:

Yes. I think that with my experience, I would say that I don’t know how to manage it … Sexual assault is, in a way, a kind of shame and scary and something illegal that has happened. And you don’t want anyone to see it.

Laura internalized the societal discourse and prevailing notions of stigma and shame, well-established concepts in this field of study. She also expressed feelings of being scared, which points to the enormous emotional impact of the phenomenon of abuse. The lived life and impact of child sexual abuse go beyond cultural taboos. For many, the desire is to keep it hidden, even into adulthood, and this is valuable knowledge for those who work with individuals who have experienced child sexual abuse. The urge to encourage these individuals to share, and the urge to offer therapeutic help, may sometimes feel intrusive. For practitioners, sensitivity around what to say, in what way, which words to use, and at which time to intervene requires profound responsiveness and sensitivity.

Another participant, Lars, talked about having felt that the abuse was wrong. He explained that it was not culturally acceptable back then – nor in the present – so he shouldered the blame, even though he knew he was innocent. He carried around these culturally unaccepted experiences, captured within him:

I think mostly that it was very wrong, very shameful. Being wrong … that I remember very clearly.

Lars and most of the other participants said that they knew the abuse was wrong from the very first time they were exposed to it. The temporal aspect appears to be intertwined – inexorably linked to memory and feelings connected to the situation. Their narratives often point to feelings of guilt that took hold within them at a very early age. However, Sara explained how she first realized that she was being abused, that it was horrible, and that the public information and discourses did not correspond with what the perpetrator was telling her. She was confused and reflected around the need to find ways of [in her words] ‘surviving’:

I don’t think I realized it until I Googled it. It was weird and horrible and stuff like that. But when you’ve been told since you were five or six years old that it’s completely normal, and that it’s a good thing and such, so, you trust it in a way.

… What I felt then, it was that kind of survival. That I had to sort something out. I had to figure things out. I had to survive. I did have to figure out how to put up with everything, in a way.

The cultural context in which the participants were a part, the household in which they were brought up, and the relationships in which they were entangled all point to material, generational, and relational enactment. Sara’s main focus was on survival, as she navigated the challenges of living with the experiences of abuse and physical assault. This can serve as a reminder to professionals who work with those exposed to child sexual abuse. The importance of recognizing these individuals’ need for agency and to be in control – rather than feeling pushed or engaged in a battle for survival – should be acknowledged. Their attachment to the perpetrator as they were growing up – dependent upon the adults around them – became distinct parts of their notions of abuse. The sexual abuse was also conceptualized by most of the participants through their therapeutic experiences, and thus, they used a therapeutic language in their meaning-making. Una explained how this played out in the present time, how she connected her past in her future through her triggers [in her words], and how she prepared for and anticipated the future by trying to overcome overwhelming emotions and memories (triggers):

I’m working a bit on identifying triggers. It can be a smell, but I don’t think about it there and then. So, it can be a smell, a perfume – a male cologne. Then a word can be said … and then I think it’s easy for me to go back ‘there’. I have come so far that I can reflect now, so I can think then it was a smell that actually triggered it.

Una conceptualized the notions of abuse through her therapeutic language, something that appeared to help most of the participants place the experiences in a psychological, cultural, and contextual framework. Furthermore, the participants reflected on their childhood abuse, which occurred in the past but continued to have an impact in the present, in their current lives. Like several other participants within the group, it is as though they unconsciously endeavoured to repress their memories, keeping them at a safe distance, and noticeably, the temporal aspect appears to be vital. Their ways of conceptualizing around their experiences of child sexual abuse constituted central aspects in their ways of knowing – captured foremost in their bodies and in their minds, as part of their life narratives.

## Discussion

Child sexual abuse needs to be understood in light of the public discourse and through individuals’ ways of knowing (Cooper, 2009; Etherington, 2004). Thus, the three overall narratives highlighted in this article speak to the many layers and intricacies concerning the phenomenon, which constitute valuable knowledge for professionals who interact with these individuals, as will be unpacked below. This project aimed to contribute with knowledge for state health and welfare institutions: to improve the quality of ‘being’ for children and adolescents who interface with these institutions.

Despite robust research on child sexual abuse, the literature has not yet fully captured a cohesive representation of the complexity concerning the lived lives of those having had these experiences. This study therefore adds to the fairly recently highlighted need for a life course perspective and emphasizes the concept of agency from the lived experience of child sexual abuse. The analysis highlights the participants’ narratives as encompassing personal and societal levels of meaning, regarding their experiences with child sexual abuse.

According to Varpanen (2019), the tension between the individual and the social can be seen in an important debate that concerns the ontological status of children’s agency. On the one hand, agency can be seen as the capacity of an individual or something existing in the individual. On the other hand, agency can be seen as emerging in social interactions – as it is located in the relations between individuals (Sugarman & Sokol, 2012) – and also through the environment they inhabit. The concept of agency emerged in the retrospective narrative analysis and was recognized for its potential impact on the participants’ lives as adults, when they interact with social and health care institutions. The three narratives elicited from the interviews in this study show how these experiences were interwoven relationally, materially, contextually, generationally, and emotionally; thus, the findings should be understood holistically. Indeed, they illustrate a complex interplay between individual, embodied, relational, contextual, and cultural factors concerning the lived life of child sexual abuse.

The beliefs that humans, especially children, are agents who can be taught to be ethical and relational to safeguard their world overlook the fact that they are entangled with an agentic, non-human world (Varpanen, 2019). Additionally, the tension between children as agentic yet developmentally unable to recognize or articulate their experience contributes to their inability to seek help contemporaneously. Research on child sexual abuse under-acknowledges the multifaced, tacit, and embodied dimensions, leaving the literature without a full picture of these deeply personal and often devastating events – events that are also often ‘unspeakable’ (Herland, 2022a, 2022b) even years later – and thus, more knowledge is needed for the field of practice. This is especially important, since research reveals that mental health nurses feel discomfort when working with individuals who have experienced child sexual abuse; the nurses report having a lack of knowledge, confidence, and preparation concerning inquiring about and responding to such a sensitive topic (Kennedy et al., 2021). Seeking help does not always feel supportive, and terms such as ‘healing’ or ‘recovery’ can be perceived as negative, as they are seen as placing the responsibility on the individual and reducing people to one-dimensional selves (Krayer et al., 2015).

The first narrative illustrates the importance for participants as adults to take control of their memories and process of recollection, which could be seen as an act of agency – in contrast to their childhood, when they were bounded to their perpetrator, relationally entangled, and their own choices or possibilities were limited. This finding speaks to professionals who need to be cautious and sensitive as to when and how to intervene.

The second narrative emphasizes the participants’ perception of grooming – their characterizations of it, such as feeling trapped or groomed, primarily relationally – but also in terms of materiality: the places and spaces in which they were entangled, such as being caught on a boat or finding themselves in an unsafe place, with no viable way to escape the abuse as ontological becomings (Spyrou et al., 2018; Varpanen, 2019). These insights have value for practitioners, especially in terms of understanding agency. The participants have diverse experiences with feeling trapped whilst in a helping relationship, and addressing this can be a delicate task. The practitioner should thus maintain an awareness of the physical conditions, environment, and sensation of restraint, as they interact with prior experiences of losses of agency.

The third narrative displays the participants’ overall perception of child sexual abuse, which reflects the cultural discourses with which they negotiate. They frequently adopted therapeutic language to explain these experiences. As noted, the analysis emphasizes the participants’ lived experiences of child sexual abuse. To argue that these participants, as children, could make their own decisions rather than be coerced by the adults who abused them – to consider them as autonomous beings – may be highly problematic (Weldemariam & Wals, 2020; Spyrou et al., 2018). These participants, as children, were intricately linked relationally, enacting a very different ontology: one which considers them as developmental beings, that is, as relational becomings (Spyrou et al., 2018; Spyrou, 2019). The narrative approach that was utilized in this study enabled an exploration of the participants’ perceptions. In this regard, the current study’s findings thus hold relevance for practice, emphasizing the importance of also being sensitively attuned to individuals’ agency as adults, which will be discussed further below.

## Recommendations: Learning From Lived Experiences

This study emphasizes and highlights the importance of increased awareness and sensitivity around the participants’ own views of child sexual abuse. Their ways of speaking about perhaps sometimes unspeakable issues constitute valuable knowledge for professionals in the fields of health care and social work practice who confront this phenomenon. The findings highlight several ways to enhance practice: for example, understanding how to provide suitable and compassionate assistance.

Child sexual abuse is a severe form of child abuse that leads to physical, mental, and emotional challenges which can have devastating and irreversible effects on survivors’ lives (Sathyamurthi & Nanditha, 2023). Adults who have experienced child sexual abuse are at a higher risk of developing physical and psychiatric disorders, particularly because the trauma they endured was severe (Felitti, 1991, 2019). However, the long-term effects of child sexual abuse can be difficult to fully grasp, comprehend (Sathyamurthi & Nanditha, 2023), or interact with. The narrative approach has allowed us to delve into the multiple layers involved and to come closer to understanding how the participants experienced their lives. The current study’s findings thus have specific implications for practice, which will be discussed below.

## Implication for Practice

The study findings demonstrate the importance that health and social work practitioners are responsive to and respectful of survivors’ lived experiences of sexual abuse. If professionals are sensitive and responsive to children’s and young people’s embodied stories, including their bodily hesitations and ambivalences, their individuality and unique processes, and their emotional load, this may represent a strong place from which to start. However, this may be a complicated task. For example, professional participants in one study (Albaek et al., 2020) described intense negative reactions in themselves when child abuse was addressed during assessments and investigations; it may also trigger anxiety, which underlines the need for profound sensitivity and training. Professionals who work with this target group need increased awareness regarding these issues, both in educational settings and in practice.

How, then, can professionals best assist individuals and families who have had sexual abusive experiences? This calls for further discussions concerning the complex issues and dilemmas involved, and also highlighting the need for a reflexive practice. I encourage studies that involve child welfare services and health care professionals as to how they can engage sensitively with children and families around the suspicion of child sexual abuse, or in treatment/recovery processes, whilst remaining attuned to their own approaches. I also advocate for further research related to the practitioner’s role in helping settings but with a primary focus on learning from the individuals who have experienced child sexual abuse as to how they view these support settings.

## Conclusion

The qualitative analysis in this study identified three central narratives, which emerge as significant findings in this article: the perception of memories, the perception of grooming, and the perception of the lived lives of child sexual abuse survivors. Findings suggest that the narratives elicited from the participants included current views but also past experiences and anticipation about the future, encompassing individual and societal levels of meaning. These narratives are entangled and inexorably linked – relationally, temporally, culturally, generationally, and materially. The study findings offer essential insights into the complex dimensions surrounding the lived experiences of child sexual abuse, which can significantly aid professionals who work with this vulnerable population.

## Limitations

This study’s sample of 14 participants, comprising more women than men, could be viewed as a limitation. The study was retrospective, and it may be seen as problematic to use adults’ knowledge to enhance understanding of children’s experiences of child sexual abuse. Nonetheless, as the participants’ reflections contribute valuable knowledge regarding the meaning-making around their experiences and narratives through a retrospective lens, the article should be read accordingly (Herland, 2022a, 2022b, 2023). This study is one contribution to the field of social and health care practitioners working with children and families facing the complex phenomenon of child sexual abuse. However, I encourage further research: for example, studying the dialogue between survivors and professionals more in-depth, including the professionals’ approaches and their reflections and reflexivity around the phenomena of abuse.
